# MicroRNA-378 is involved in hedgehog-driven epithelial-to-mesenchymal transition in hepatocytes of regenerating liver

**DOI:** 10.1038/s41419-018-0762-z

**Published:** 2018-06-18

**Authors:** Jieun Kim, Jeongeun Hyun, Sihyung Wang, Chanbin Lee, Youngmi Jung

**Affiliations:** 1 Department of Integrated Biological Science, College of Natural Science, Pusan National University, Pusan, 46241 Korea; 2 Department of Biological Sciences, College of Natural Science, Pusan National University, Pusan, 46241 Korea

## Abstract

Healthy livers have a remarkable regenerative capacity for reconstructing functional hepatic parenchyma after 70% partial hepatectomy (PH). Hepatocytes, usually quiescent in normal healthy livers, proliferate to compensate for hepatic loss after PH. However, the mechanism of hepatocyte involvement in liver regeneration remains unclear. Hedgehog (Hh) pathway plays an important role in tissue reconstitution by regulating epithelial-to-mesenchymal transition (EMT) in liver disease. MicroRNA (miRNA) is involved in cell proliferation and differentiation during embryonic development and carcinogenesis. It was recently reported that miR-378 inhibits transdifferentiation of hepatic stellate cells into myofibroblasts by suppressing Gli-Krüppel family member 3 (Gli3), the Hh-target gene. We hypothesized that miR-378 influences EMT in hepatocytes by interfering with Hh signaling during liver regeneration. As hepatocytes were highly proliferative after PH in mice, miR-378 and epithelial marker, Ppar-g or E-cadherin were downregulated, whereas both Hh activators, Smoothened (Smo) and Gli3, and the EMT-inducing genes, Tgfb, Snail and Vimentin, were upregulated in the regenerating livers and in hepatocytes isolated from them. Compared to cells with or without scramble miRNA, primary hepatocytes transfected with miR-378 inhibitor contained higher levels of Gli3 with increased expression of the EMT-promoting genes, Tgfb, Snail, Col1a1, and Vimentin, suggesting that miR-378 influenced EMT in hepatocytes. Smo-depleted hepatocytes isolated from PH livers of Smo-flox mice showed downregulation of EMT-promoting genes and Gli3, with upregulation of miR-378 and E-cadherin compared to Smo-expressing hepatocytes from PH liver. In addition, delivery hepatocyte-specific AAV8 viral vector bearing Cre recombinase into Smo-flox mice impeded EMT in Smo-suppressed hepatocytes of PH liver, indicating that Smo is critical for regulating hepatocyte EMT. Furthermore, the application of miR-378 mimic into mice with PH delayed liver regeneration by interrupting hepatocyte EMT. In conclusion, our results demonstrate that miR-378 is involved in hepatocyte EMT by regulating Hh signaling during liver regeneration.

## Introduction

The liver has remarkable regenerative ability, first described in Greek mythology and more recently proven in studies of rodents undergoing two-thirds partial hepatectomy (PH). Liver regeneration after PH proceeds in an orderly fashion without necrosis and inflammation; therefore, murine models with surgical manipulation of PH are widely used for studying the biological processes of liver regeneration^1^. Surgical removal of two-thirds of the liver leads to a hyperplastic response in the remaining hepatic tissue until the original liver mass is reconstituted^2^. Liver architecture and function are also retained during liver regeneration^3^. Mature hepatocytes, which are quiescent and rarely divide in normal healthy livers, re-enter the cell cycle and replicate to compensate for the loss of liver mass^2,4^. Along with these hepatocytes, residual cholangiocytes and hepatic stellate cells (HSCs) are believed to mediate the repopulation of liver cells after PH^5,6^. However, it is poorly understood how the remnant liver regenerates the various types of liver cells.

Epithelial-to-mesenchymal transition (EMT) is a biological process in which epithelial cells gradually lose their adherence and apical-basal polarized phenotype, while gaining the migratory and invasive properties of mesenchymal cells^7^. There is altered expression of some epithelial (e.g. E-cadherin, Zona occludens-1, and Cytokeratin) and mesenchymal (e.g., Snail, Vimentin, α-Smooth muscle actin, and ColIa1) genes during this process^8^. In chronically damaged livers, hepatocytes undergo EMT to escape destruction by TGFb, presenting the proliferative mesenchymal phenotype of myofibroblasts (MFs)^9^. It was recently reported that hepatocytes have transiently decreased levels of hepatocyte nuclear factor 4 alpha, a marker of mature hepatocytes, as well as increased expression of EMT-promoting factors such as Yes-associated protein 1, and Gli-Krüppel family member 2 (Gli2), during the post-PH replicative period^10^. These findings indicate the transdifferentiation of hepatocytes during liver regeneration, but it is unclear how hepatocyte fate is regulated in this process.

Hedgehog (Hh) signaling is known to orchestrate tissue reconstruction and to be involved in EMT in damaged livers^11–13^. In chronically or severely injured livers, Hh signaling promotes the transition of quiescent HSCs and immature ductular cells into MFs^13–16^. Although mature hepatocytes in healthy adult livers are non-responsive to Hh signaling, hepatocytes in damaged livers activate it^11,17^. In particular, increased activation expression of the Hh ligands, Sonic Hh (Shh) and Indian Hh, and the Hh-target genes, Gli1 and Gli2, is observed in the hepatocytes of regenerating livers after 70% PH^11^. In PH-treated mice with suppression of Hh signaling, liver regeneration is significantly inhibited, with reduced hepatocyte proliferation, decreased EMT, and disrupted matrix reconstitution, leading to reduced survival rates^11^. These findings suggest that Hh signaling is associated with proliferation and/or transdifferentiation of hepatocytes during post-PH liver regeneration.

MicroRNAs (miRNAs) have been implicated in cell proliferation and differentiation during embryonic development and cancer progression^18,19^. Several miRNAs, such as miR-101 and miR-181a, are reported to be involved in TGFb–induced EMT of hepatocytes in liver cirrhosis and hepatocellular carcinoma^20,21^. However, it remains unclear how and what kinds of miRNAs are involved in the hepatocyte reprogramming during normal liver regeneration. We recently demonstrated that miR-378 is involved in HSC transition in the fibrotic liver by interacting Hh signaling^22^. Given that Hh signaling is associated with the fate-change of hepatocytes and HSCs in the liver-repair process, it is possible that miR-378–mediating Hh signaling influences the EMT of hepatocytes in regenerating livers after PH. We investigated whether and how miR-378 is involved in hepatocyte transition during this process. We found that reduced miR-378 leads to the activation of Hh signaling which is involved in EMT in regenerating liver. Overexpression of miR378 impedes liver regeneration in mice by suppressing hepatocyte EMT. Taken together, these findings suggest miR-378 as an important regulator for hepatocyte transdifferentiation in regenerating livers.

## Results

### Expressional changes of miR-378 with Hh-target genes in regenerating livers

To generate the experimental model of adult normal-liver regeneration, we performed 70% PH on wild-type (WT) mice, who were sacrificed 1, 3, 6, 12, 24, 48, 72, or 96 h post-surgery. The liver weight (LW) and LW-to-body weight (BW) ratio (LW/BW) began to be elevated at 48 h, increasing by almost 2.3-fold at 96 h compared with livers at 0 h post-PH (Supplementary Figure S1A). Histologic and enzymatic damage, assessed with hematoxylin and eosin (H&E) staining and serum ALT/AST analysis, respectively, was observed in post-PH livers for 24 h, but the injuries significantly recovered by 48 h post-PH (Supplementary Figures S1B&C). In addition, immunostaining with Ki67, a marker of cell proliferation, showed the accumulation of Ki67-positive hepatocytes at 24 h. The number of these cells sharply increased at 48 h and was maintained until 96 h (Supplementary Figure S2). These results confirmed that the murine PH model was successfully generated.

Since the EMT process occurs during liver regeneration after PH^5^, we analyzed the expression of EMT-related genes in liver tissues. These levels in the regenerating liver were compared with those in resected liver tissue at each post-PH time point. Expression of *Tgfb*, EMT-promoting factor^23,24^, immediately increased 1 h after PH and declined to nearly basal levels at 6 h. And then, *Tgfb* began to be upregulated at 48 h, and then rapidly downregulated at 96 h. The mRNA expression of *Snail, TGFb-*regulated transcription factors promoting EMT^23^, was immediately raised 1 h after PH, peaking at almost 9-fold above basal levels at 6 h before being rapidly downregulated to nearly basal levels at 12 h. Thereafter, *Snail* transiently increased and decreased, returning to basal levels at 96 h. Expression of *Vimentin*, a mesenchymal marker, was maintained at the basal level until 12 h, then increased significantly until 96 h. Meanwhile, *Ppar-g* and *E-cadherin*, EMT inhibitor^25^, were downregulated at 6 h at which point *Snail* expression was higher, and showed steadily low levels until it was restored to nearly its basal level at 72 h (Supplementary Figure S3A).

Given that Hh signaling orchestrates EMT in the liver-repair process^5,13^ and that miR-378 targeting GLI3 influenced the transition of HSCs, with expression regulated by SMO^22^, we examined the expression of miR-378, *Smo*, and *Gli3* in regenerating livers. *Smo* was slightly upregulated at 3 h, rapidly downregulated at 6 h, and then tended to increase gradually until 96 h. MiR-378 inhibited by SMO revealed the opposite expression pattern with *Smo*. MiR-378 showed lowered expression at 3 h, then began to elevate, peaking at 12 h, then gradually decreasing until 96 h. Expression of *Gli3* targeted by miR-378 greatly increased at 6 h, similar to the pattern of *Smo*, and declined to below basal levels at 12 h, the point at which miR-378 was highly expressed. It then gradually increased until 72 h (Supplementary Figure S3B). These findings indicate that EMT occurs in regenerating liver and SMO-GLI3-miR-378 axis is associated with this process.

### Hepatocytes underwent EMT in regenerating livers after PH

**Fig. 1:**
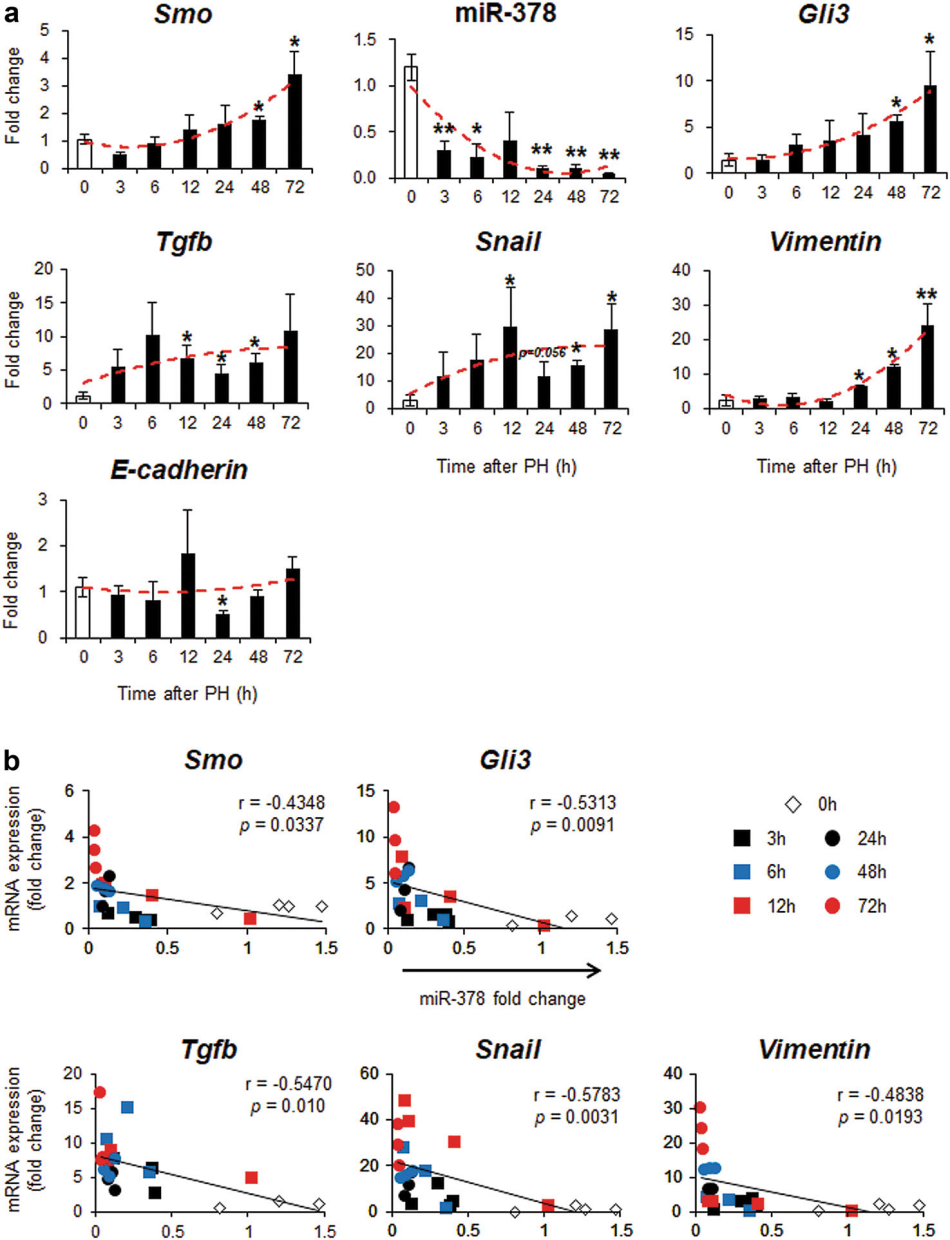
Expressional changes of miR-378, Hh target genes, and EMT activator in hepatocytes from mice with PH. **a** Expression of *Smo*, miR-378, *Gli3*, *Tgfb, Snail*, *Vimentin, and E-cadherin* was assessed by qRT-PCR analysis in primary hepatocytes isolated from mice at 3, 6, 12, 24, 48 and 72 h after PH. All results of relative expression values are shown as mean ± s.e.m. of triplicate experiments (unpaired two-sample Student’s *t* test, *p < 0.05 **p < 0.005 vs. 0 h). **b** Pearson’s correlation coefficient between miR-378 expression and *Smo, Gli3, Tgfb, Snail*, and *Vimentin* (*N* = 24, Pearson’s correlation *r* = −0.435, *p* = 0.034 with *Smo*; *r* = −0.531, *p* = 0.009 with *Gli3*; *r* = −0.547, *p* = 0.01 with *Tgfb;* *r* = −0.578, *p* = 0.003 with *Snail*; *r* = −0.484, *p* = 0.019 with *Vimentin*)

**Fig. 2:**
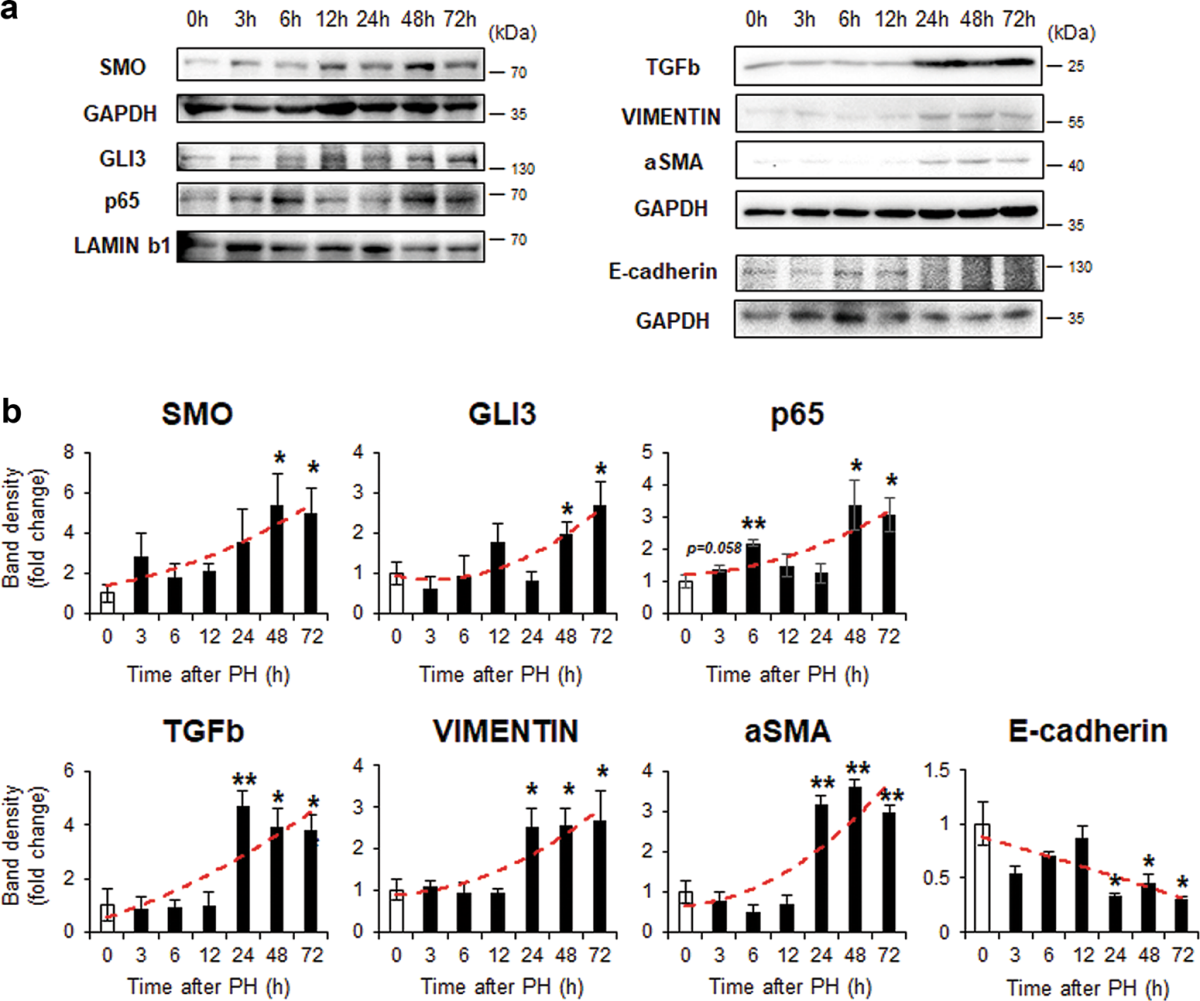
Protein levels of Hh activators and EMT-related genes in hepatocytes from PH livers **a** Western blot analysis for SMO (86 kDa), nuclear GLI3 (145 kDa) and p65 (65 kDa), processed form of TGFb (25 kDa*)*, VIMENTIN (57 kDa), αSMA (42 kDa*)*, E-cadherin (120 kDa), GAPDH (36 kDa), and LAMINb1 (68 kDa) in primary hepatocytes isolated from PH livers of mice. GAPDH and LAMINb1 was used as an internal control. Immunoblots shown represent one of three independent experiments with similar results. **b** Cumulative densitometric analyses of SMO, GLI3, p65, TGFb, Vimentin, αSMA, and E-cadherin western blots are displayed as the mean ± s.e.m. (unpaired two-sample Student’s *t* test, *p < 0.05 **p < 0.005 vs. 0 h)

When hepatocytes are treated with specific triggers, such as TGFb, hepatitis C viral protein, or organochlorine pesticides, they undergo an EMT/MET-like process^24,26–29^. Emerging evidence also supports a hepatocyte-mediated EMT-like process in human liver disease and murine liver fibrosis^30–32^. Given that miR-378 influences GLI3 expression in the liver repair process^22^ and the expressional changes of EMT-related and Hh-target genes were observed in PH livers, we investigated whether hepatocytes undergo an EMT and whether miR-378-mediated Hh signaling contributes to this process. Hepatocytes were isolated from PH livers to evaluate hepatocyte-specific changes in gene expression during liver regeneration. Compared with primary hepatocytes isolated from quiescent livers (0 h), *Smo* mRNA levels in hepatocytes from PH given mice were maintained at basal levels until 12 h, then began to increase, showing significant upregulation at 48 and 72 h. MiR-378 expression showed consistent reduction, below 40% of basal levels, whereas the expression of *Gli3* targeted by miR-378^22^ tended to increase gradually, with significant upregulation at 48 and 72 h. Expression of *Tgfb* and *Snail* gradually increased and showed the plateau after presenting the significant elevation at 12 h. *Vimentin* was upregulated at 24 h, peaking at 72 h, while *E-cadherin* was downregulated at 24 h and restored to nearly its basal level at 48 h (Fig. 1a). MiR-378 expression was inversely correlated with the Hh-target genes, *Smo* and *Gli3*, and the EMT-related genes, *Tgfb, Snail* and *Vimentin*, in hepatocytes from mice PH livers (Fig. 1b). SMO protein levels and the nuclear form of Gli3 were significantly elevated at 48 h and maximized at 72 h, supplementary their RNA expression in hepatocytes. Because p65 was reported to be activated by Smo and to inhibit miR-378 transcription^22^, p65 expression was examined. Expression of p65 in nuclear form was low before PH but raised 3 h after PH, and gradually declined at 12 h. Thereafter p65 sharply increased at 48–72 h, the time when SMO expression is maximal. The protein expression of TGFb, aSMA, and VIMENTIN, also showed the plateau after presenting the significant increase at 24 h. E-cadherin gradually decreased after PH, and rarely detected post 24 h, showing the opposite expressional pattern with the expression of mesenchymal markers (Fig. 2a, b). In addition, hepatocytes from PH liver at 48 and 72 h had the remarkably increased migratory capability compared with cells from nonPH liver, as assessed by wound-healing assay (Supplementary Figure S4). The results suggested that hepatocytes undergo EMT after PH, and Hh activators and miR-378 are associated with this process during liver regeneration.

### MiR-378 suppressed hepatocyte EMT

We found that hepatocytes underwent EMT and the higher level of miR-378 in quiescent hepatocytes was significantly downregulated in hepatocytes undergoing EMT. Hence, it is possible that miR-378 prevents hepatocytes from undergoing EMT. To verify this hypothesis, we suppressed miR-378 in quiescent hepatocytes and assessed the expression of EMT-related genes in these cells. AML12, a murine cell line of normal hepatocytes, was transfected with miR-378 inhibitor or scrambled miR as negative control (NC). Compared to miR-378 expression in AML12 cells treated with or without the NC, its level was successfully reduced, followed by elevated expression of *Gli3* in AML12 cells with miR-378 inhibitor at 12 or 24 h. Levels of mesenchymal markers, *Tgfb*, *Snail*, *Vimentin* and *Col1a1* were significantly elevated in miR-378 inhibitor-treated AML12 cells, whereas the levels of epithelial markers, including *E-cadherin* and *Zo-1* were significantly alleviated in these cell, compared with two control groups (Supplementary Figure S5).

**Fig. 3:**
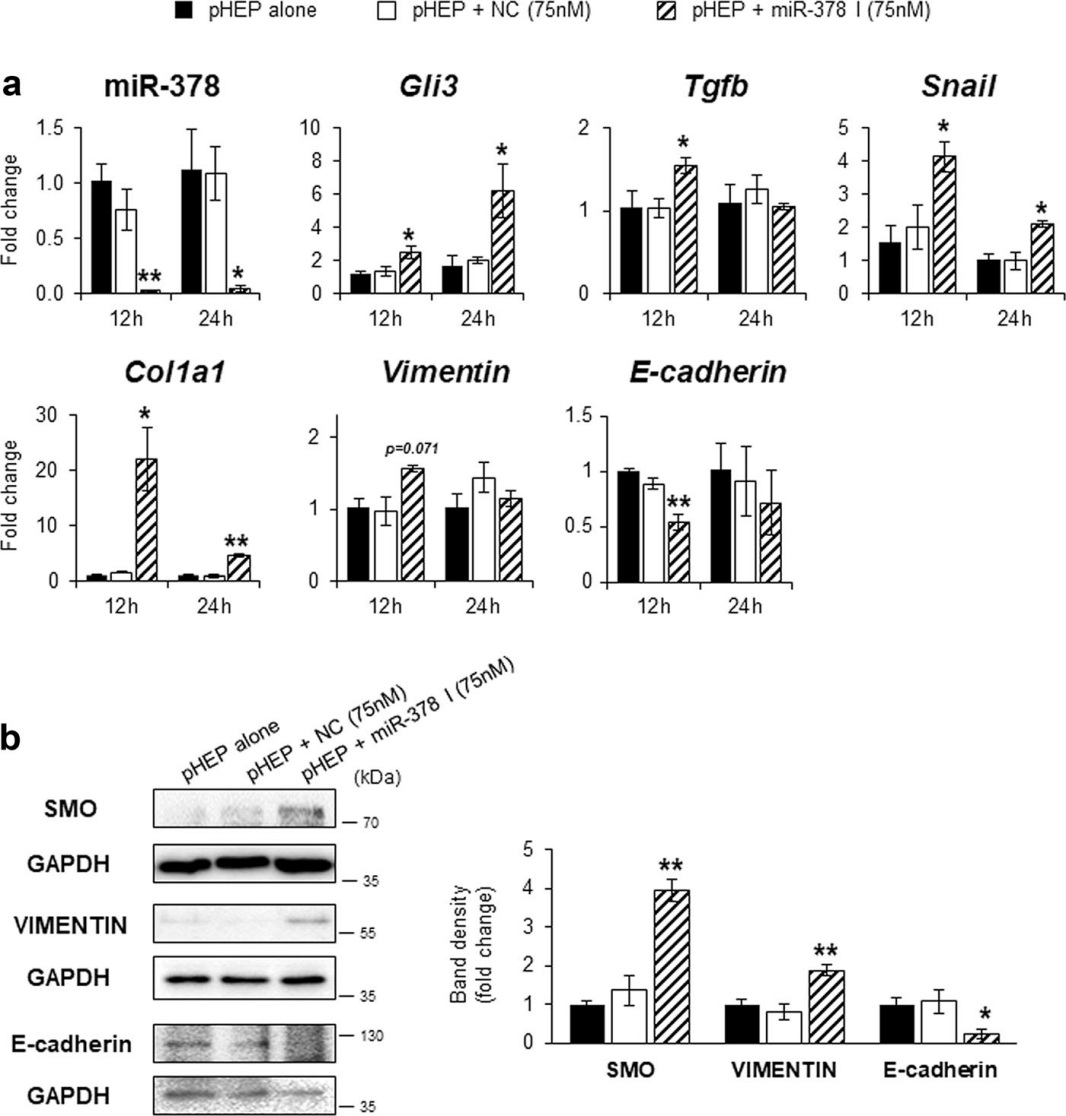
MiR-378 inhibitor promotes EMT in primary hepatocytes of mice. **a** qRT-PCR of miR-378, *Gli3*, *Tgfb*, *Snail*, *Col1a1, Vimentin* and *E-cadherin* in primary hepatocytes (pHEP), which were isolated from WT mice and transfected with miR-378 inhibitor (I, 75 nM, diagonal lined bar) or scramble miR (NC, 75 nM, white bar) for 12 and 24 h. All results of relative expression values are shown as mean ± s.e.m. of triplicate experiments (unpaired two-sample Student’s *t* test, *p < 0.05 **p < 0.005 vs. both cells cultured alone (black bar) and cultured with NC). **b** Western blot analysis and cumulative densitometric analyses for SMO, VIMENTIN, E-cadherin, and GAPDH in these transfected cells for 24 h. GAPDH was used as an internal control. Immunoblots shown represent one of three independent experiments with similar results. Results are displayed as the mean ± s.e.m. (unpaired two-sample Student’s *t* test, *p < 0.05 **p < 0.005 vs. both cells cultured alone (black bar) and cultured with NC)

Since primary cells better reflect the physiological state of cells in vivo compared to cell lines, we isolated hepatocytes primarily from livers of WT mice and transfected them with miR-378 inhibitor or scramble miR. The mRNA levels of *Gli3*, *Tgfb*, *Snail*, *Col1a1*, and *Vimentin* were upregulated following miR-378 reduction in miR-378 inhibitor-treated hepatocytes, compared to primary hepatocytes treated with or without the NC (Fig. 3a). The mRNA levels of *E-cadherin* were downregulated in miR-378 inhibitor-treated hepatocytes, compared to other groups. Protein data also confirmed the RNA data by presenting higher expression of SMO and VIMENTIN and lower expression of E-cadherin in miR-378 suppressed hepatocytes than the other groups (Fig. 3b). These data demonstrate that miR-378 is involved in hepatocyte EMT by suppressing Gli3 expression.

### Smo plays the major role in the miR-378-mediated EMT in hepatocytes

**Fig. 4:**
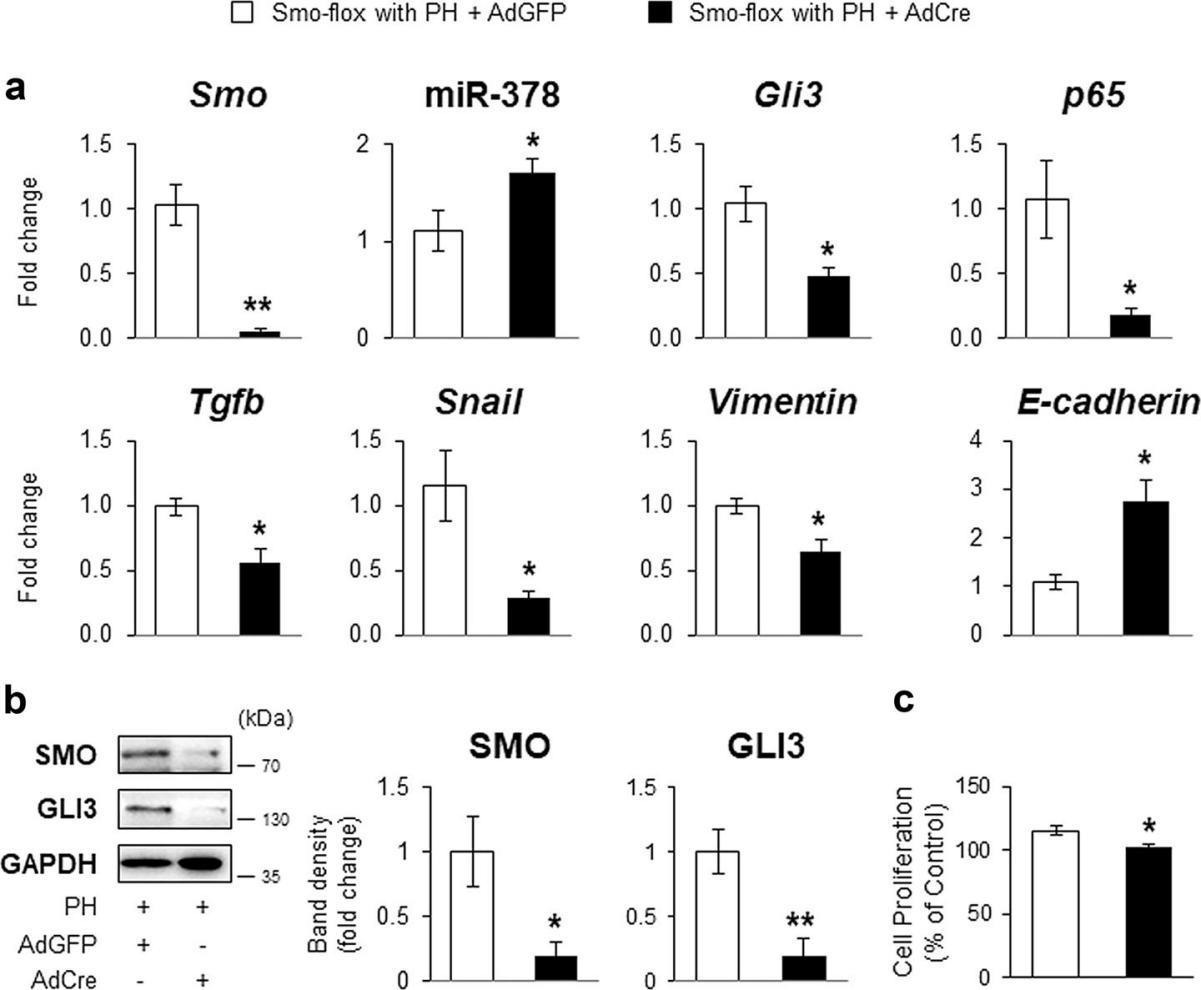
Abrogating Hh signaling in hepatocytes disrupts EMT in hepatocytes. **a** qRT-PCR for *Smo*, miR-378, *Gli3*, *p65, Tgfb*, *Snail*, *Vimentin*, and *E-cadherin* in primary hepatocytes. These cells were isolated from Smo-flox transgenic mice with PH (Smo-flox with PH) at 48 h, and then transfected with adenoviruses containing GFP (AdGFP: white bar) or Cre recombinases (AdCre: black bar) for 24 h. All results of relative expression values are shown as mean ± s.e.m. of triplicate experiments (unpaired two-sample Student’s *t* test, *p < 0.05, **p < 0.005 vs. Smo-flox with PH + AdGFP). **b** Western blot and cumulative densitometric analysis for SMO and active form of GLI3 in these cells. GAPDH was used as an internal control. Representative images from triplicated experiments with similar results were shown. Results are displayed as the mean ± s.e.m. (unpaired two-sample Student’s *t* test, *p < 0.05, **p < 0.005 vs. Smo-flox with PH + AdGFP). **c** Cell proliferation was measured by MTS assays in these cells. The mean ± s.e.m. results obtained from three independent experiments are graphed (unpaired two-sample Student’s *t* test, *p < 0.05, **p < 0.005 vs. Smo-flox with PH + AdGFP)

Hepatocytes undergo EMT in both the specific culture conditions and in damaged human and murine livers^9,21,28,31–33^. Hh signaling plays an important role in EMT^12,16,34^. Our data also indicate that miR-378 is associated with EMT in hepatocytes of PH livers. Given that miR-378 was shown to be regulated by SMO^22^, we investigated whether Smo-mediated miR-378 was involved in hepatocyte EMT. Because Smo expression began to be upregulated significantly in hepatocytes of PH liver at 48 h, hepatocytes were isolated from Smo^tm2Amc^/J (Smo-flox) mice at 48 h after PH. And then, we conditionally depleted the Smo allele by transducing adenoviruses harboring Cre recombinase (AdCre). Adenoviruses carrying the GFP gene (AdGFP) were employed as the transfection control. Hepatocytes isolated from quiescent liver also were transfected with AdCre or AdGFP, to assess the effect of adenovirus transfection in quiescent liver. The RNA levels of *Smo*, miR-378*, Gli3*, and EMT-markers, were similar between these two groups of cells from quiescent liver (Supplementary Figure S6A). Cell proliferation was not different between these two groups. However, it was significantly elevated in the AdGFP-transfected hepatocytes from PH livers compared with the adenovirus-transfected hepatocytes from quiescent livers (Supplementary Figure S6B). After confirming that the transfection hardly impacted on the expression of these genes in hepatocytes from quiescent livers, we compared the gene expression in adenovirus-transfected hepatocytes from PH livers of Smo-flox mice. EMT-favorable genes, including *Smo*, *Gli3*, *p65, Tgfb, Snail*, and *Vimentin* were significantly downregulated, whereas the EMT-inhibiting genes, miR-378 and *E-cadherin*, were significantly upregulated in AdCre-treated hepatocytes from Smo-flox mice with PH (black bar), compared to the AdGFP-treated hepatocytes from Smo-flox mice with PH (white bar) (Fig. 4a). The protein levels of SMO and GLI3 supported the RNA expression data, with a significant increase in SMO and GLI3 in Smo-expressing hepatocytes compared to Smo-deleted cells from PH livers (Fig. 4b). Smo-suppressed hepatocytes also showed the significantly reduced cell proliferation compared with Smo-expressing cells (Fig. 4c).

**Fig. 5:**
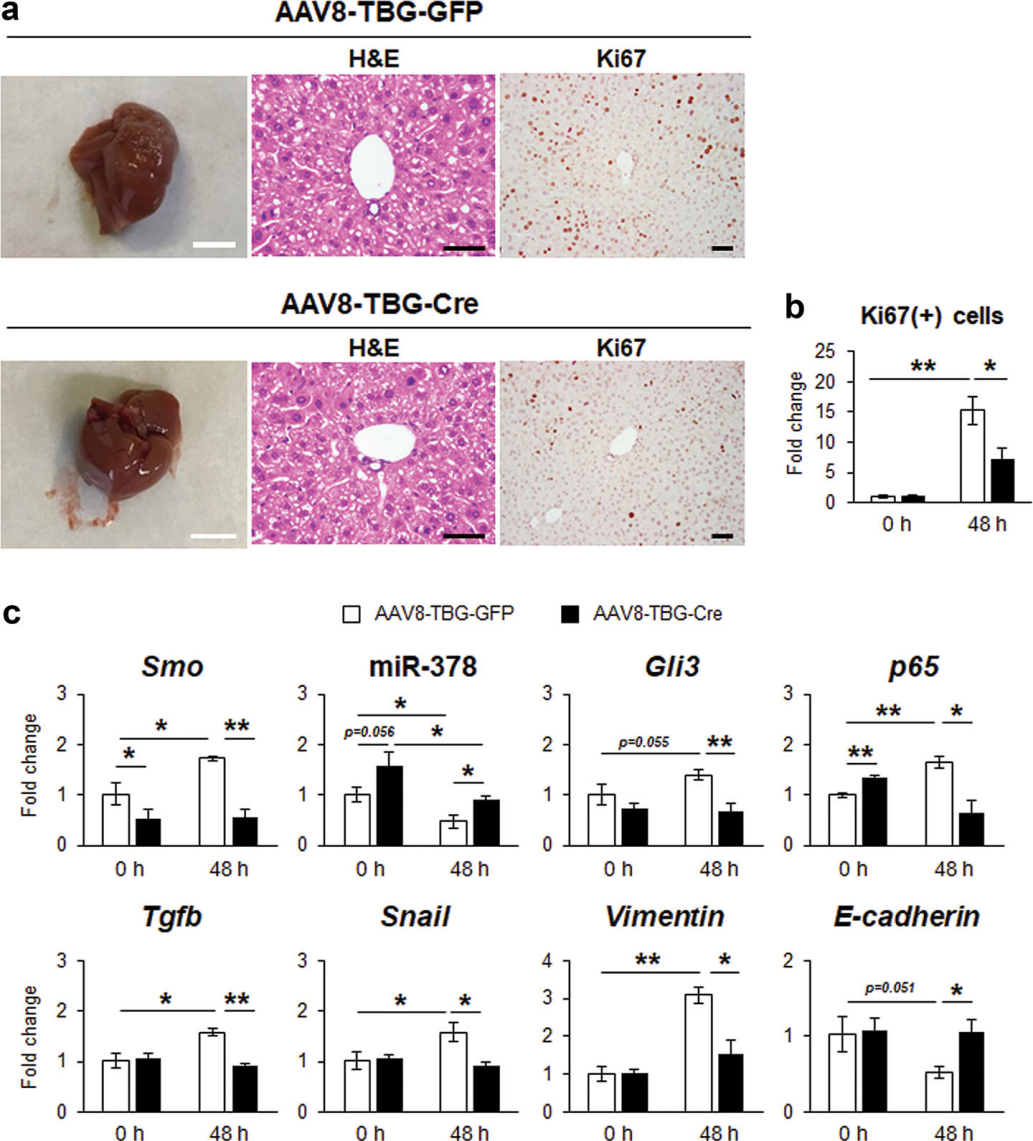
Targeted deletion of Smo in livers inhibits hepatocyte proliferation and EMT after PH. **a** Images, H & E-, and Ki67-stained sections of livers from AAV8-TBG-GFP or AAV8-TBG-Cre-treated mice at 48 h after PH (*N* = 4 per group). Representative images of PH liver at 48 h were shown (liver image: scale bar = 50 mm/H&E and Ki67 stain: scale bar = 50 µm). **b** Quantitative Ki67-stained data from these mice. Ki67-positive hepatocytic cells were quantified by counting the total number of Ki67-positive cells per field. Mean ± s.e.m. results are graphed (one-way ANOVA with Tukey corrections, *p < 0.05 **p < 0.005) (**c**) qRT-PCR for *Smo*, miR-378, *Gli3*, *p65*, *Tgfb*, *Snail*, *Vimentin* and *E-cadherin* in these livers. All results of relative expression values are shown as mean ± s.e.m. of triplicate experiments (one-way ANOVA with Tukey corrections, *p < 0.05 **p < 0.005)

To investigate the effect of Smo on EMT in vivo, Smo-flox mice were injected with a hepatocyte-tropic AAV8 viral vector bearing Cre recombinase (AAV8-TBG-Cre) to delete Smo specifically in hepatocytes. AAV8 bearing GFP (AAV8-TBG-GFP) was used as a control vector. After viral injection, mice underwent PH and we examined liver response at 48 h post PH. Compared with AAV8-TBG-GFP-treated mice, regenerating liver remnants of AAV8-TBG-Cre-treated mice were relatively small and contained less Ki67-positive cells (Fig. 5a, b), although they did not show the significant change in LW and LW/BW post PH (Supplementary Figure S7). Serum levels of ALT/AST were higher in AAV8-TBG-Cre than the AAV8-TBG-GFP mice post PH (Supplementary Figure S7). The upregulated *Smo, Gli3, p65, Tgfb, Snail*, and *Vimentin*, and the downregulated miR-378 and *E-cadherin* in AAV8-TBG-GFP-treated PH liver were reversed in AAV8-TBG-Cre-treated PH liver, presenting the impaired EMT in PH liver with Smo suppression (Fig. 5c).

**Fig. 6:**
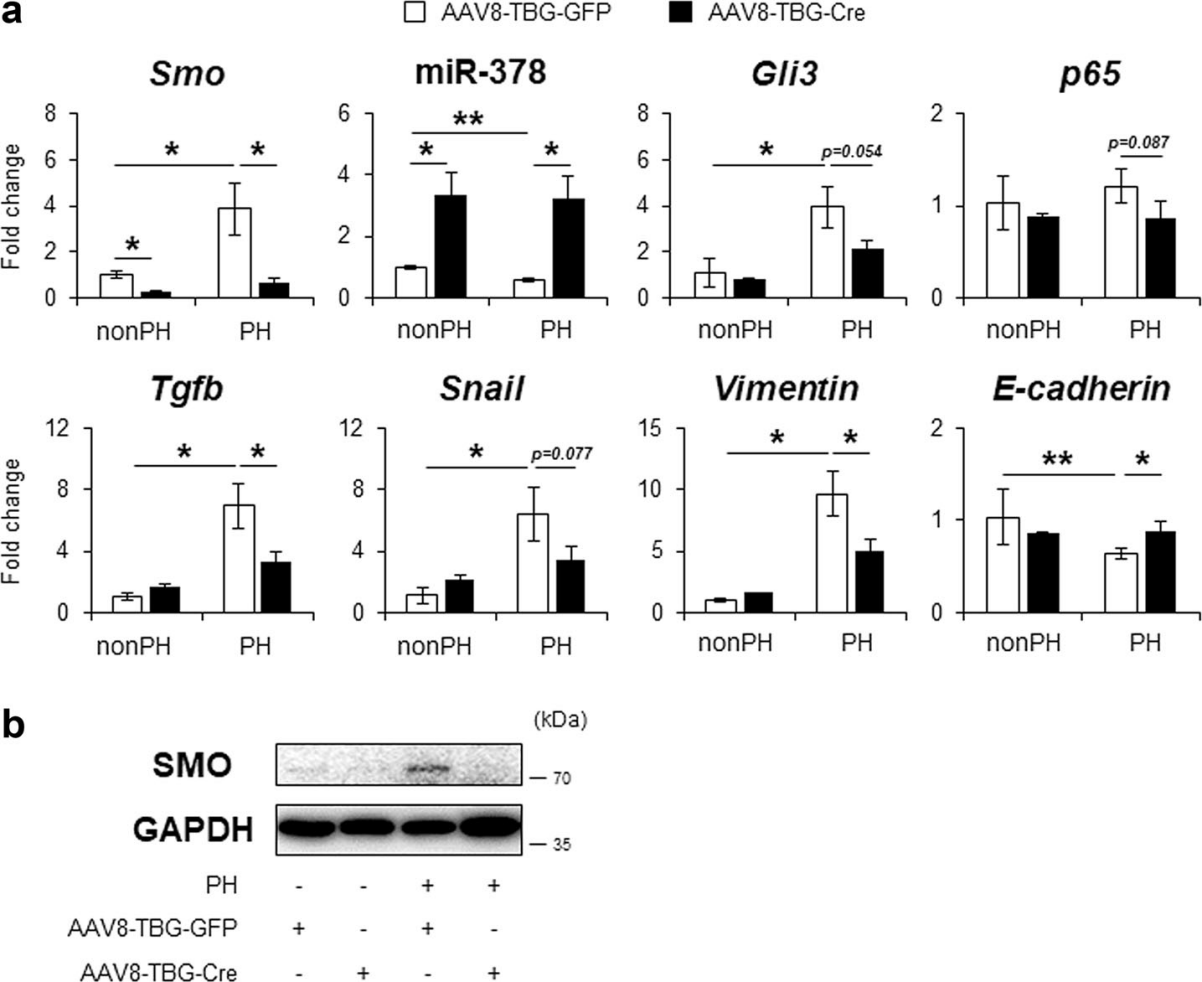
Targeted disruption of Smo in hepatocytes suppressed EMT after PH. **a** qRT-PCR for *Smo*, miR-378, *Gli3*, *p65*, *Tgfb*, *Snail*, *Vimentin* and *E-cadherin* in primary hepatocytes, which were isolated from AAV8-TBG-GFP or AAV8-TBG-Cre-treated mice with (PH) or without PH (nonPH) (*N* = 3 per group). Cells from PH livers were obtained from livers at 48 h post PH. All results of relative expression values are shown as mean ± s.e.m. of triplicate experiments (one-way ANOVA with Tukey corrections, *p < 0.05 **p < 0.005). **b** Western blot analysis for SMO (86 kDa) and GAPDH (36 kDa) in primary hepatocytes isolated from these mice. GAPDH was used as an internal control. Immunoblots shown represent one of three independent experiments with similar results

To confirm whether hepatocyte-specific deletion of Smo influenced the impeded EMT in PH livers of AAV8-TBG-Cre-treated mice, primary hepatocytes from these mice and were analyzed for EMT-related gene expression. Increased expressions of *Smo, Gli3, p65, Tgfb, Snail*, and *Vimentin* and decreased expressions of miR-378 *and E-*cadherin in hepatocytes from AAV8-TBG-GFP-treated PH livers were reversed in cells from AAV8-TBG-Cre-treated PH livers (Fig. 6a). Interestingly, regardless of PH, *Smo* expression was lower and miR-378 level higher in the AAV8-TBG-Cre than the AAV8-TBG-GFP group (Figs. 5 & 6). SMO protein was rarely detected in hepatocytes from AAV8-TBG-Cre-treated mice, supporting that Cre successfully delivered into and specifically removed Smo in hepatocytes (Fig. 6b). Taken together, these results demonstrate that SMO is a master regulator of EMT in hepatocytes by altering miR-378 expression during liver regeneration.

### MiR-378 hinders liver regeneration in PH liver

**Fig. 7:**
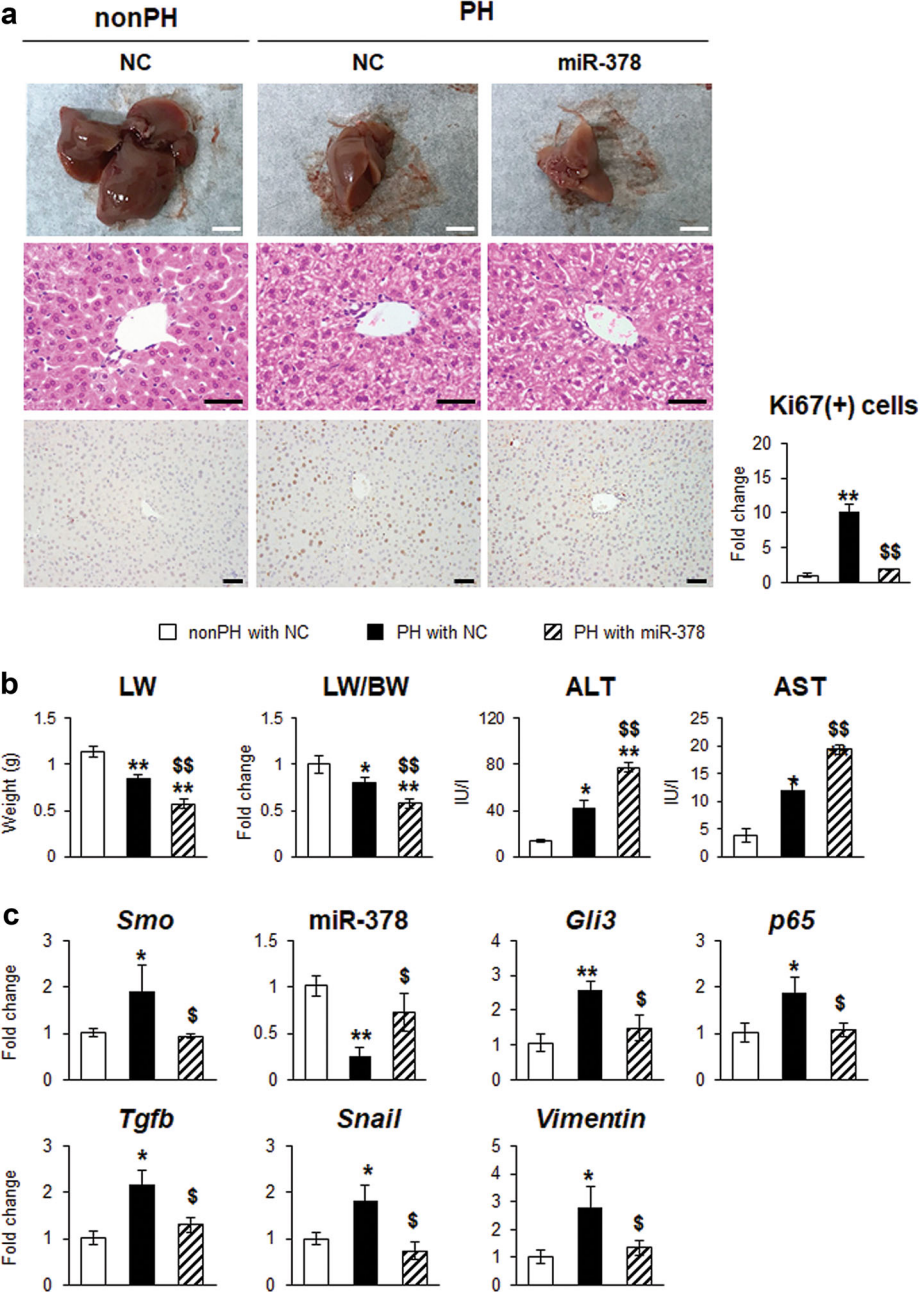
Liver regeneration is impaired in miR-378 mimic-transfected mice. **a** Images, H & E-, and Ki67-stained sections of livers from miR-378 mimic- or scramble RNA- (negative control: NC) transfected mice with (*N* = 5 per group) or without PH (*N* = 3 per group). Representative images of quiescent livers (nonPH) and PH liver at 72 h were shown (top panel: scale bar = 50 mm/middle and bottom panel: scale bar = 50 µm). Ki67-positive hepatocytic cells were quantified by counting the total number of Ki67-positive cells per field. Mean ± s.e.m. results are graphed (one-way ANOVA with Tukey corrections, *p < 0.05, **p < 0.005 vs. nonPH with NC, $*p* < 0.05 $$*p* < 0.005 vs. PH with NC). **b** LW, LW/BW, and serum ALT and AST levels of each groups were graphed as mean ± s.e.m (one-way ANOVA with Tukey corrections, *p < 0.05, **p < 0.005 vs. nonPH with NC, $*p* < 0.05 $$*p* < 0.005 vs. PH with NC). **c** qRT-PCR for *Smo*, miR-378, *Gli3*, *p65*, *Tgfb*, *Snail*, and *Vimentin* in livers of these mice. All results of relative expression values are shown as mean ± s.e.m. of triplicate experiments (one-way ANOVA with Tukey corrections, *p < 0.05, **p < 0.005 vs. nonPH with NC, $*p* < 0.05 $$*p* < 0.005 vs. PH with NC)

To investigate the function of miR-378 on liver regeneration in vivo, miR-378 mimic or NC was injected via i.p. route of administration into mice, which were subjected to PH. Because most downregulation of miR-378a, upregulation of other genes and cell proliferation were observed in both livers and hepatocytes at 72 h post-PH in our analysis, we assessed the expression of miR-378 and EMT-related genes at this time point. Images of regenerating liver remnant after PH clearly showed that the miR-378 mimic-treated liver was relatively small compared with NC-treated liver. Serum levels of ALT/AST and the accumulation of Ki67-positive cells greatly increased in PH liver, compared with quiescent liver with NC. However, these enzymatic levels were significantly higher and Ki67-expressing cells were less obvious in miR-378 mimic-treated liver than two control groups (Fig. 7a, b). The LW and LW/BW also significantly declined in miR-378 treated mice with PH compared with two control groups of mice. The upregulated *Smo, Gli3, p65, Tgfb, Snail*, and *Vimentin*, and the downregulated miR-378 in PH liver with NC were reversed in PH liver with miR-378 mimic, presenting the alleviation of these Hh-target and EMT-related genes, and the elevation of miR378 (Fig. 7).

**Fig. 8:**
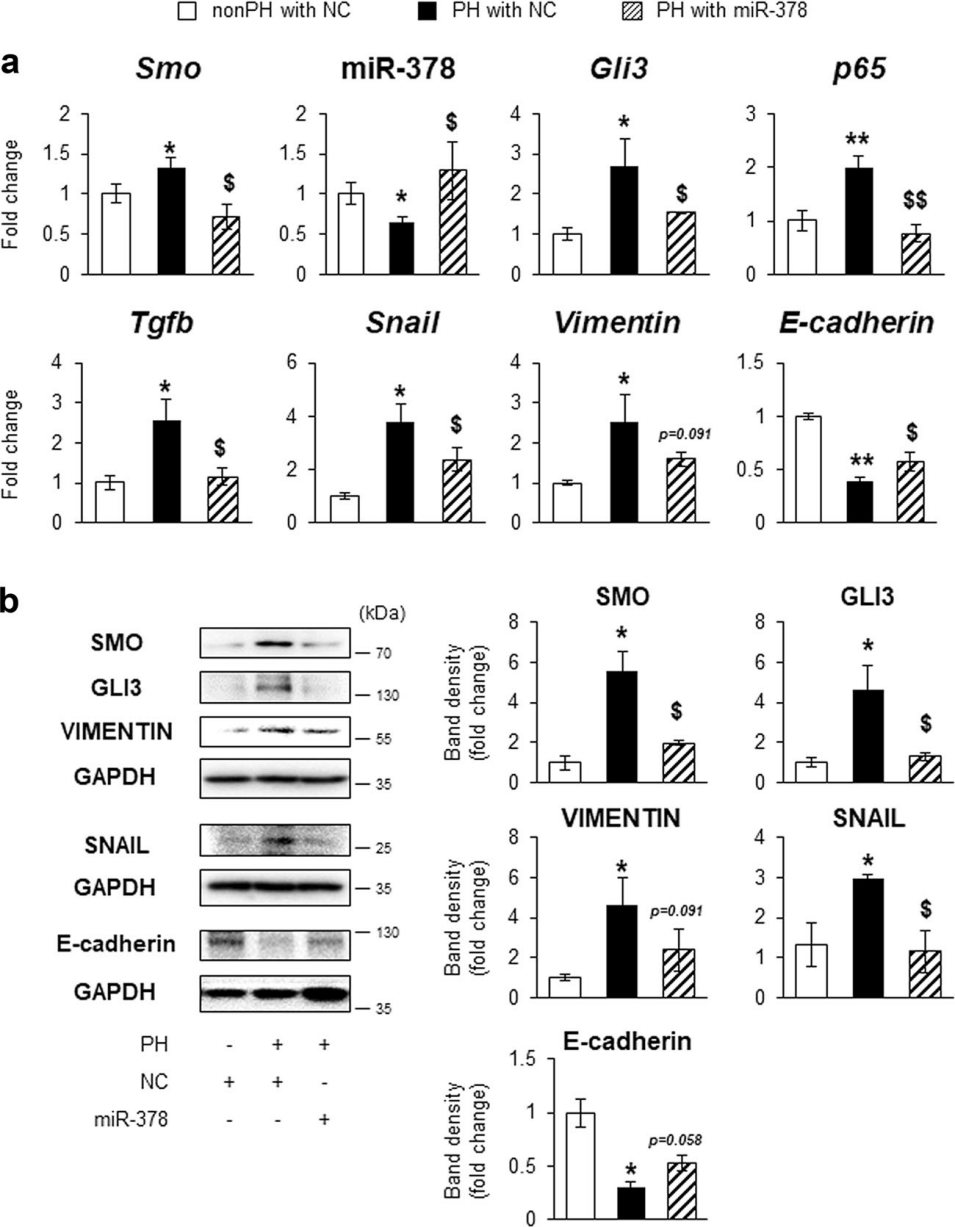
MiR-378 suppresses hepatocyte EMT in mice with PH. **a** qRT-PCR for *Smo*, miR-378, *Gli3*, *p65*, *Tgfb*, *Snail*, *Vimentin*, and *E-cadherin* in hepatocyte which were isolated from PH or quiescent liver and transfected with miR-378 mimic (PH with miR378: diagonal lined bar) or NC (nonPH with NC: white bar/ PH with NC: black bar). All results of relative expression values are shown as mean ± s.e.m. of triplicate experiments (one-way ANOVA with Tukey corrections, *p < 0.05 **p < 0.005 vs. nonPH with NC, $*p* < 0.05 $$*p* < 0.005 vs. PH with NC). **b** Western blot and cumulative densitometric analyses for SMO (86 kDa), GLI3 (145 kDa), VIMENTIN (57 kDa), SNAIL (29 kDa), E-cadherin (120 kDa), and GAPDH (36 kDa) in these cells. GAPDH was used as an internal control. Immunoblots shown represent one of three independent experiments with similar results. Results are displayed as the mean ± s.e.m. (*p < 0.05, **p < 0.005 vs. nonPH with NC, $*p* < 0.05 $$*p* < 0.005 vs. PH with NC)

To assess whether the impeded liver regeneration in miR-378 mimic-treated mice resulted from the suppressed EMT in hepatocytes, the levels of miR-378 and EMT-related genes were examined in hepatocytes isolated from these mice. In line with data of gene expression in PH liver transfected with miR-378 or NC, primary hepatocytes from NC-treated mice with PH at 72 h had the higher level of *Smo, Gli3, p65, Tgfb, Snail*, and *Vimentin*, and the lower level of miR-378 and *E-cadherin* than hepatocytes from NC-treated mice without PH. Their expressional changes were reverse in hepatocytes from miR-378 mimic-transfected PH liver, displaying the downregulation of Hh-activators and EMT-promoting genes and the upregulation of miR-378 and *E-cadherin* compared with NC-transfected hepatocytes from PH livers (Fig. 8a). Protein analysis also confirmed the RNA data by presenting decreased expression of SMO, GLI3, VIMENTIN and SNAIL and increased expression of E-cadherin in hepatocytes from miR-378 mimic-treated PH liver compared with cells from NC-treated PH liver (Fig. 8b). Taken together, these results suggest that miR-378 is involved in liver regeneration by regulating EMT in hepatocytes.

## Discussion

Many studies have reported that hepatocytes undergo EMT and acquire migratory capacity under specific EMT-inducing culture conditions^24,26,27,30,35,36^. A previous study also showed that hepatocytes experienced intrinsic dedifferentiation in collagen monolayer cultures with upregulation of mesenchymal markers, including N-cadherin, Vimentin, and Col1a1, although there was no change in the expression of Snail and E-cadherin, unlike the TGFb–mediated EMT response^37^. Although it remains controversial whether EMT occurs in response to liver damage in vivo, growing evidence suggests hepatocyte EMT in injured human and murine livers^21,31,32^. Albumin-expressing hepatocytes were positive for fibroblast-specific protein 1 in carbon tetrachloride (CCl_4_)-induced murine liver fibrosis^30^. In hepatitis B virus-infected human livers, hepatocytes were double-positive for transferrin, a hepatocyte marker, and for collagen or Snail^31^. Using the lineage tracing technique, Zeisberg et al. suggested hepatocytes-undergoing EMT as a source of MFs, leading to liver fibrosis in vivo^30^. Reduced liver fibrosis in CCl_4_-treated mice with Snail-deleted hepatocytes supports EMT in hepatocytes in vivo^32^. Nuclear factor erythroid 2-related factor, an important regulator of antioxidant system, has been shown to regulate EMT in renal and pulmonary fibrosis^38,39^ and be essential for liver regeneration in PH liver^40^. In addition, our findings demonstrated that hepatocytes undergo EMT and the Hh signaling-mediated miR-378 is involved in this process during liver regeneration of PH-treated mice.

Although miR-378 expression was inversely correlated with expression of Smo and Gli3 in collected liver remnants at various times during liver regeneration, downregulation of miR-378 in hepatocytes from PH livers was observed earlier than increase in Smo, suggesting that other factors might be involved in the miR-378 reduction. Prior work demonstrated that p65 activated by SMO-suppressed miR-378 expression in HSCs, contributing to HSC activation and liver fibrosis^22^. As p65 is a subunit of nuclear factor kappa B(NF-κB)^41^, it is possible that NF-κB signaling activators, such as tumor necrosis factor-α, also participate in increasing p65 expression during hepatocyte transition. Hence, we examined whether p65 induced the earlier reduction of miR-378 in hepatocytes from PH liver. We found that nuclear form of p65 increased in hepatocytes from PH liver at 3 h (Fig. 2). The RNA level of p65 was also inversely correlated with miR-378 expression in hepatocytes from PH liver at various times (*r* = −0.469, *p* = 0.024), although p65 RNA tended to be upregulated at 3 h and began to be raised significantly at 24 h (data not shown).

Hh signaling regulated EMT in epithelial liver cells, such as hepatocytes and cholangiocytes, contributing to the fibrotic repair response in patients with primary biliary cirrhosis or nonalcoholic steatohepatitis (NASH). This was also demonstrated in murine models of bile duct ligation-induced biliary fibrosis or diet-induced NASH^13,34^. In the present study, we showed that the expression of EMT-related genes was remarkably lower in Smo-deleted hepatocytes from PH mice than in Smo-expressing hepatocytes from PH mice, suggesting that the canonical Hh pathway activated through SMO plays an important role in hepatocyte EMT during liver regeneration after PH. In addition, p65 levels declined, followed by upregulation of miR-378 and downregulation of GLI3 in Smo-deleted hepatocytes (Fig. 4), indicating that SMO is an essential factor linking miR-378 with the Hh signaling pathway in promoting EMT. Hyun et al^17^. reported the accumulation of GLI3-positive hepatocytic cells and elevated protein levels of GLI3 with sustained fibrosis in the livers of CCl_4_-treated rats, whereas these were both significantly alleviated in the restored livers of rats transplanted with human chorionic plate-derived mesenchymal stem cells after CCl_4_ exposure. These results support that GLI3 expression influences fibrogenesis by regulating hepatocyte EMT. Therefore, our findings demonstrate that hepatocytes experience EMT during liver regeneration and miR-378 is involved in this process through interfering with the Hh pathway, particularly the SMO-GLI3 axis.

However, upregulation of EMT markers, TGFb, Snail, and Vimentin, in hepatocytes was observed earlier than increases in SMO and GLI3. Growing evidence suggest that the MF-HSCs impact hepatocyte reprogramming during liver regeneration^5,10,42^. HSCs become activated almost immediately after PH and these activated HSCs predominantly secret a number of factors, including TGFb^42^. Subsequently, TGFb induces quiescent mature hepatocyte to undergo EMT^26,43,44^. In line with these findings, HSC activation-related signaling factors, such as TGFb and Smo, were upregulated in liver at 1 and 3 h post PH, respectively. Therefore, it is possible that the HSCs are first activated by TGFb and/or Hh signaling pathway, and these activated HSCs trigger hepatocyte EMT by stimulating TGFb, which contributes to activating Hh signaling. In concert with TGFb signaling, the Hh signaling seems to amplify EMT in hepatocytes. However, further studies are needed to investigate the interaction of TGFb with Hh signaling in EMT of hepatocytes during liver regeneration.

## Materials and methods

### Experimental animals

C57BL/6 wild-type (WT) mice (Hyochang, Dae-gu, Korea) and Smo^tm2Amc^/J (Smo-flox) mice (Jackson Laboratory, Bar Harbor, ME, USA) were maintained in appropriate animal facilities at Pusan National University. All mice were housed with a 12-h light/dark cycle and had free access to food and water. Animal care and surgical procedures were carried out according to the provisions of the National Institutes of Health (NIH) guidelines for the Care and Use of Laboratory Animals. The animal protocol used in this study was approved by the Pusan National University Institutional Animal Care and Use Committee (PNU-IACUC) for ethical procedures and scientific care (Approval Number PNU-2016-1193).

70% PH was performed on 8–10-week-old male C57BL/6 WT mice (*N* = 46) under isofluorane anesthesia, according to the method of Higgins and Anderson^45^. The procedures were performed between 8 a.m. and 12 p.m. All animals resumed normal activities after recovery from anesthesia and were killed at 1 h (*N* = 6), 3 h (*N* = 6), 6 h (*N* = 5), 12 h (*N* = 6), 24 h (*N* = 6), 48 h (*N* = 5), 72 h (*N* = 6), or 96 h (*N* = 6) after PH. Resected quiescent livers were used as 0 h comparisons and the regenerating liver remnants were formalin-fixed or snap-frozen in liquid nitrogen for subsequent analysis.

Male Smo-flox mice at 7-week-old were injected by tail vein with 1.5 × 10^11^ genome copies of AAV8-TBG-GFP (*N* = 10) or AAV8-TBG-Cre (*N* = 10) (Vector Biolabs, Rockville, MD, USA) to selectively delete the Smo gene in hepatocytes. Five days after viral injection, mice underwent PH and were sacrificed at 48 after PH to obtain liver tissue or primary hepatocytes (*N* = 7/each group). Resected quiescent livers were used as 0 h comparisons.

To examine the effect of miR-378 in vivo, in vivo-jetPEI (Polyplus Transfection, Illkirch, France) was used according to the manufacturer’s protocols. 8-week-old male C57BL/6 WT mice were injected intraperitoneally (i.p.)with 6 nmol per mice of miR-378 mimic (*N* = 5) or scramble miRNA (*N* = 5) (Bioneer) as a negative control 12 h before PH. Mice without undergoing PH (*N* = 3) were injected with scramble miRNA at the same time with PH group.

### Isolation of primary hepatocytes and cell culture

Hepatocytes were isolated from livers of WT or Smo-flox mice as described previously^10^. Briefly, mice were anesthetized with Zoletil50 (5 mg kg^−1^ body weight, Virbac S.A, France) to immobilize them in the recumbent position on a treatment table, and the inferior vena cava was cannulated under aseptic conditions. Livers were perfused *in situ* with EGTA and collagenase (Roche, Indianapolis, IN, USA) to disperse the cells. Primary hepatocytes were separated from nonparenchymal cells using Percoll density gradient centrifugation. As determined by Trypan Blue exclusion, cell viability was >92% in all experiments. Primary hepatocytes were cultured on collagen-coated 6-well plates at a density of 3 × 10^5^ cells per well in Williams’ Medium E without phenol red (Sigma-Aldrich, St. Louis, MO, USA), supplemented with 5% fetal bovine serum (FBS; Gibco, Thermo Fisher Scientific, Waltham, MA, USA), 1 μM dexamethasone, and a cocktail solution of penicillin/streptomycin (P/S), ITS + (insulin, transferrin, selenium complex, BSA, and linoleic acid), GlutaMAX™, and HEPES (Gibco). After cell attachment (~4–6 h after plating), the culture medium was replaced with serum-free Williams’ Medium E containing 0.1 μM dexamethasone, and a cocktail solution of P/S, ITS+, GlutaMAX™, and HEPES (Gibco) for hepatocyte maintenance.

AML12 (CRL-2254; ATCC), a cell line of normal mouse hepatocytes, was cultured on 6-well plates at a density of 3 × 10^5^ cells per well in Dulbecco’s Modified Eagle Medium (DMEM; Gibco) supplemented with 10% FBS and 1% P/S at 37 °C in a humidified atmosphere containing 5% CO_2_.

### Cell transfection

To inhibit the expression of miR-378 in both primary hepatocytes and AML12, cells were transfected with 75 nM or 100 nM of miR-378 inhibitor (AccuTarget™ mouse miRNA-378a-3p inhibitor, Bioneer), respectively, using Lipofectamine RNAi/MAX transfection reagent (Invitrogen, Thermo Fisher Scientific) according to the manufacturer’s instructions. Equal concentrations of scrambled miRNA (miRNA inhibitor negative control #1, Bioneer) were used as negative controls. After 12 h, the medium was replaced with fresh medium, and the transfected cells were incubated at 37 °C in a 5% CO_2_ atmosphere for 12 or 24 h.

For adenoviral transduction of primary hepatocytes isolated from Smo-flox mice with or without surgical liver resection (48 h post-PH), cells were cultured overnight in serum-depleted medium before treatment. The adenoviruses harboring either the GFP gene (AdGFP; Vector Biolabs) or the Cre recombinase gene (AdCre; Vector Biolabs) were added to primary hepatocytes at a multiplicity of infection (MOI) of 80, as described previously^46^. Virus-containing medium was aspirated and replaced with fresh medium at 24 h post-transfection. The efficiency of adenovirus infection was determined by >95% GFP-positive cells after transfection (data not shown).

### RNA analysis

Total RNA was extracted from liver tissues or cells with TRIzol reagent (Ambion, Thermo Fisher Scientific). The concentration and purity of RNA were determined using a nanodrop. Template cDNA was synthesized from total RNA using the SuperScript First-strand Synthesis System (Invitrogen) or miScript Reverse Transcriptase Kit (Qiagen, Valencia, CA, USA) according to the manufacturer’s protocols. We performed the real-time quantitative reverse transcriptional polymerase chain reaction (qRT-PCR) analysis by using the Power SYBR Green Master Mix (Applied Biosystems, Thermo Fisher Scientific) or the miScript SYBR Green PCR Kit (Qiagen) according to the manufacturer’s specifications (Eppendorf, Mastercycler Real-Time PCR). All reactions were triplicated and the data were analyzed according to the ∆∆C_t_ method. The 40 S ribosomal protein S9 (RPS9) mRNA for mRNA and the U1A small nuclear RNA (RNU1A) for miRNA were used to normalize expression levels. The sequences of all primers used in this study are summarized in Supplementary Table S1. All PCR products were directly sequenced for genetic confirmation (Macrogen, Seoul, Korea).

### Western blot

To extract total proteins, cells were homogenized in triton lysis buffer (TLB) and centrifuged at 13000 rcf for 15 min. The supernatants containing whole protein extract were used in subsequent biochemical analysis. To separate the nuclear and cytosolic fractions, cells were homogenized and suspended in buffer A (10 mM HEPES, 50 mM NaCl, 1 mM DTT, 0.1 mM EDTA, 0.1 mM PMSF) with protease inhibitors (Roche) and incubated on ice for 20 min. After adding 0.1% NP-40, the lysates were incubated for an additional 20 min. After centrifugation at 5000 × *g* for 2 min, the supernatant was collected for the cytosolic fraction. The pellets were resuspended with buffer B (20 mM HEPES, 400 mM NaCl, 1 mM DTT, 1 mM EDTA, 1 mM PMSF, 1 mM EGTA) and incubated on ice for 30 min. After centrifugation at 13,000 r.p.m. for 15 min, the supernatant was saved for the nuclear fraction. The supernatants containing cytosolic and nuclear protein extracts were used in subsequent biochemical analyses. Protein concentration was measured with a Pierce BCA Protein Assay Kit (Thermo Scientific). Equal amounts of protein lysates were separated by 8 or 10% SDS-PAGE and then transferred onto polyvinylidene difluoride (PVDF) membranes (Millipore, Darmstadt, Germany). Rabbit anti-Smo antibody (diluted 1:1000; Abcam), rabbit anti-Gli3 antibody (diluted 1:1000; Abcam), rabbit anti-p65 antibody (diluted 1:2000; Santacruz) rabbit anti-Vimentin antibody (diluted 1:1000; Santacruz), mouse anti-aSMA antibody (diluted 1:1000; Abcam), rabbit anti-TGFb antibody (diluted 1:1000; Cell Signaling technology), mouse anti-E-cadherin (diluted 1:500; BD bioscience), rabbit anti-Snail (diluted 1:1000; Santacruz) were used as the primary antibodies. Mouse anti-GAPDH antibody (diluted 1:1000; AbD Serotec., Oxford, UK) and rabbit-anti-Lamin β1 antibody (diluted 1:1000; Abcam) were used as internal controls for cytosolic and nuclear proteins, respectively. Horseradish peroxidase (HRP)-conjugated anti-rabbit or anti-mouse IgG (Amersham ECL™, GE Healthcare, Milwaukee, WI, USA) was used as the secondary antibody. Protein bands were detected using an EzWestLumi ECL solution (ATTO Corporation, Tokyo, Japan) per the manufacturer’s specifications (ATTO Corporation, Ez-Capture II). Protein-band density was measured using CS Analyzer software (Version 3.00.1011, ATTO & Rise Corporation). Band density of each target protein was normalized to the density of the own loading control.

### Liver histology and immunohistochemistry

Liver specimens were fixed in 10% neutral buffered formalin (NBF; Sigma), embedded in paraffin and cut into 4 µm sections. The specimens were deparaffinized, hydrated, and stained with H&E to examine hepatic morphology.

For immunohistochemistry, liver sections were deparaffinized, hydrated, and incubated in 3% hydrogen peroxide to block endogenous peroxidase. Antigen retrieval was performed by heating in 10 mM sodium citrate buffer (pH 6.0) for 10 min in a microwave. The specimens were then blocked in Protein Block solution (Dako, Carpinteria, CA, USA) for 30 min at room temperature (RT) followed by incubation with primary antibody at 4 °C overnight. Other sections were also incubated at 4 °C overnight in non-immune sera. Mouse Ki67 antibody (diluted 1:2000; Novocastra, Leica Microsystems, Newcastle upon Tyne, UK) was used as a primary antibody and diluted in Protein Diluent (Dako). Polymer-HRP anti-rabbit (Dako) was used as a secondary antibody and 3,3′-diaminobenzidine (DAB) for brown color was used to visualize the protein. To quantify Ki67-positive hepatocytic cells, 10 randomly chosen 20× fields/section were evaluated by counting the total number of Ki67-stained cells/field for each mouse.

### Measurement of ALT and AST

Serum alanine transaminase (ALT/GPT, glutamate-pyruvate transaminase) and aspartate transaminase (AST/GOT, glutamate-oxaloacetate transaminase) levels were measured using GOT and GPT reagents (ASAN PHARMACEUTICAL, Seoul, Korea) according to the manufacturer’s instructions.

### Cell proliferation (MTS) assay and scratch assay

Cell proliferation was measured with a CellTiter Proliferation Assay (Promega, Madison, WI, USA) according to the manufacturer’s protocols. Briefly, cells were plated at a density of 1 × 10^4^ cells per well in 96-well plates with the indicated treatment and time. After adding the MTS reagent, the plates were incubated in a CO_2_ incubator at 37° until the color developed. Absorbance was then measured at a wavelength of 490 nm using a Glomax multi-detection system (Promega). Scratch assays were performed by growing cells to a confluent monolayer, and making a manual scratch using a 200 μL pipette tip. Then, floating cells were washed out and fresh medium was added. As the incubation time passed, the width of scratch was narrow and it was recorded by taking photographs (×20) using an Olympus inverted microscope (Olympus Optical Co., Ltd., Tokyo, Japan) from 24, 36 and 48 h after the scratch. Empty area in each time point was quantified with NIH image J version 1.49 analysis software (Rasband, W.S., ImageJ, U.S. National Institutes of Health, Bethesda, Maryland, USA, http://imagej.nih.gov/ij/, 1997–2012) and compared with that in the initiation of cell migration.

### Statistical analysis

Results are expressed as mean ± s.e.m. Statistically significant differences between the control and treatment groups or subgroups were analyzed with two-sample Student’s *t* test or one-way analysis of variance (ANOVA) followed by post hoc Tukey’s test. Differences were considered significant when *p*-values were <0.05. The degree of correlation between the expression levels of miR-378a-3p and Smo, Gli3, Tgfb, Snail, and Vimentin in primary hepatocytes isolated from PH liver was analyzed by the Pearson’s correlation coefficient. Statistical analyses were performed using SPSS (version 21.0.0.0, IBM Corp., Armonk, NY, USA).

## Electronic supplementary material

Supplementary Information (marked-up ver)
