# Supplementary material for: MicroRNA-378 is involved in hedgehog-driven epithelial-to-mesenchymal transition in hepatocytes of regenerating liver
Source: Cell Death Dis. 2018 Jun 18;9(7):721. doi: 10.1038/s41419-018-0762-z (PMC6006434; doi:10.1038/s41419-018-0762-z)

Supplementary Information

MicroRNA-378 is involved in hedgehog-driven epithelial-to-mesenchymal transition in hepatocytes of regenerating liver

Jieun Kim[1+], Jeongeun Hyun[1+], Sihyung Wang[1], Chanbin Lee[1], and Youngmi Jung[1, 2*]

[1]Department of Integrated Biological Science, [2]Department of Biological Sciences, College of Natural Science, Pusan National University, Pusan, Korea, 46241

[*]**Correspondence to**

Dr. Youngmi Jung, Department of Biological Sciences, College of Natural Science, Pusan National University, Pusandaehak-ro 63beon-gil, Geumjeong-gu, Pusan, Korea, 46241 y.jung@pusan.ac.kr; Tel.: +82-51-510-2262; Fax: +82-51-581-2962

[+] These authors contributed equally to this work.

Supplementary Figure legends

Supplementary Figure S1. Liver regeneration of mice after partial hepatectomy (PH). (a) Liver weight (LW) and relative LW to body weight (LW/BW) of male C57BL/6 mice after PH (n=5 or 6 /time group). Results were graphed as mean±s.e.m. (one-way ANOVA with Tukey corrections, *p<0.05 **p<0.005 vs 1st phase, $p<0.05 $$p<0.005 vs 2nd phase divided by broken line). (b) Hematoxylin and eosin (H&E) staining for assessing the liver morphology in representative liver sections from PHx mice (scale bar = 50 μm). (c) Serum ALT and AST levels of each groups were graphed as mean±s.e.m. (one-way ANOVA with Tukey corrections, *p<0.05 **p<0.005 vs 1st phase, $p<0.05 $$p<0.005 vs 2nd phase, #p<0.05 ##p<0.005 vs 3rd phase divided by broken line).

Supplementary Figure S2. Cell proliferation during liver regeneration after PH. (a) Immunohistochemistry for Ki67, a marker of S phase during cell cycle, in liver sections from the representative PHx mice (n ≥ 5 mice / group) (scale bar = 50 μm). (b) Quantitative Ki67-stained data from all mice. Ki67-positive cells were quantified by counting the total number of Ki67-positive cells per field. Data was presented as fold increase compared with quiescent livers (0h). Mean±s.e.m. results are graphed (one-way ANOVA with Tukey corrections, *p<0.05 **p<0.005 vs 1st phase divided by broken line).

Supplementary Figures S3. Downregulation of miR-378 with activation of Smo-Gli3 and EMT in regenerating livers of mice. (a) qRT-PCR analysis for the expression of EMT-related genes, *Tgfb, Snail, Vimentin, Ppar-g, and E-cadherin* (b) *Smo,* miR-378 and *Gli3* in all mice after PH. Results were presented as fold increase compared to own resected liver tissues and means±s.e.m. were graphed (paired two-sample Student's t-test, *p<0.05 **p<0.005 vs 0h).

Supplementary Figure S4. Increased migratory ability of hepatocytes from PH liver. (a and b) Cell migration were measured by wound healing assays in primary hepatocytes from the liver at 48h

or 72h after PH or without PH (nonPH). Artificial wounds were created on cells in confluence. Images were taken at 0, 24, 48 hours after wound. Data shown represent one of three experiments with similar results and the mean±s.e.m. of triplicate experiments are graphed (paired two-sample Student's t-test, *$p<0.05$ **$p<0.005$ vs 0h). (a: Representative images, b: Quantification of wound closure).

Supplementary Figure S5. Inhibition of miR378 induces EMT in AMl12, hepatocyte cell line. qRT-PCR of miR-378, *Gli3*, *Tgfb*, *Snai1*, *Vimentin*, *Col1a1*, *E-cadherin* and *Zo-1* in AML12 cells which were transfected with miR-378 inhibitor (I, 100nM, diagonal lined bar) or scrambled miR as a negative control (NC, 100nM, white bar) for 12 and 24 hours. All results of relative expression values are shown as mean±s.e.m. of triplicate experiments (unpaired two-sample Student's t-test, *$p<0.05$ **$p<0.005$ vs both cells cultured alone (black bar) and cultured with NC).

Supplementary Figure S6. Analysis of gene expression and proliferation of primary hepatocytes from quiescent livers of Smo-flox transgenic mice transfected with AdGFP or AdCre. (a) qRT-PCR for *Smo*, miR-378, *Gli3*, *Tgfb*, *Snai1*, *Vimentin*, and *E-cadherin* in primary hepatocytes which were isolated from Smo-flox transgenic mice without PH, and then transfected with either adenoviruses containing GFP (AdGFP: white bar) or Cre recombinases (AdCre: black bar). All results of relative expression values are shown as mean±s.e.m. of triplicate experiments (unpaired two-sample Student's t-test, *$p<0.05$, **p<0.005 vs Smo flox PH+AdGFP). (b) Cell proliferation was measured by MTS assays in primary hepatocytes which were isolated from Smo-flox transgenic mice with PH (PH) or without PH (nonPH), and then transfected with either adenoviruses containing GFP (AdGFP: white bar) or Cre recombinases (AdCre: black bar). The mean±s.e.m. results obtained from tree independent experiments are graphed (unpaired two-sample Student's t-test, *$p<0.05$, **p<0.005).

Supplementary Figure S7. Analysis of liver weight and serum ALT/AST levels in Smo-deleted mice by AAV8-TBG-Cre. LW, LW/BW, and serum ALT and AST levels of AAV8-TBG-GFP- or

AAV8-TBG-Cre-treated mice at 48 h after PH (N=4/group). Each groups were graphed as mean±s.e.m. (one-way ANOVA with Tukey corrections, *p<0.05 **p<0.005).

Supplementary Table

Supplementary Table S1. Mouse primer sequences used for qRT-PCR

Gene	Forward	Reverse
miR-378	ACTGGACTTGGAGTCAGAAGG	
U1A snRNA	CGACTGCATAATTTGTGGTAGTGG	
Smo	CAGCAAGATCAACGAGACCA	AAGTGGCAGCTGAAGGTGAT
p65	CTGATGTGCATCGGCAAG	TGCTGGGAAGGTGTAGGG
Gli3	GCAACCTCACTCTGCAACAA	CCTTGTGCCTCCATTTTGAT
Snail	TCCAGCAGCCCTACGACCAG	AGGCCGAGGTGGACGAGAA
Vimentin	GCTTCTCTGGCACGTCTTGA	CGCAGGGCATCGTTGTTC
Tgfb1	TTGCCCTCTACAACCAACACAA	GGCTTGCGACCCACGTAGTA
E-cadherin	ACCTCTGGGCTGGACCGA	CCTGATACGTGCTTGGGTTGAA
Zo-1	CCACCTCTGTCCAGCTCTTC	CACCGGAGTGATGGTTTTCT
Col1a1	GAGCGGAGAGTACTGGATCG	GCTTCTTTTCCTTGGGGTTC
Ppar-g	CACAATGCCATCAGGTTTGG	GCTGGTCGATATCACTGGAGATC
S9	CTTCATCTTGCCCTGGTCCA	GACTCCGGAACAAACGTGAGGT

Primer sequences shown in this table were used for qRT-PCR. All values were normalized to the level of RPS9 or U1A snRNA for total mRNA or miRNA, respectively.

Supplementary Figures

Supplementary Figure S1

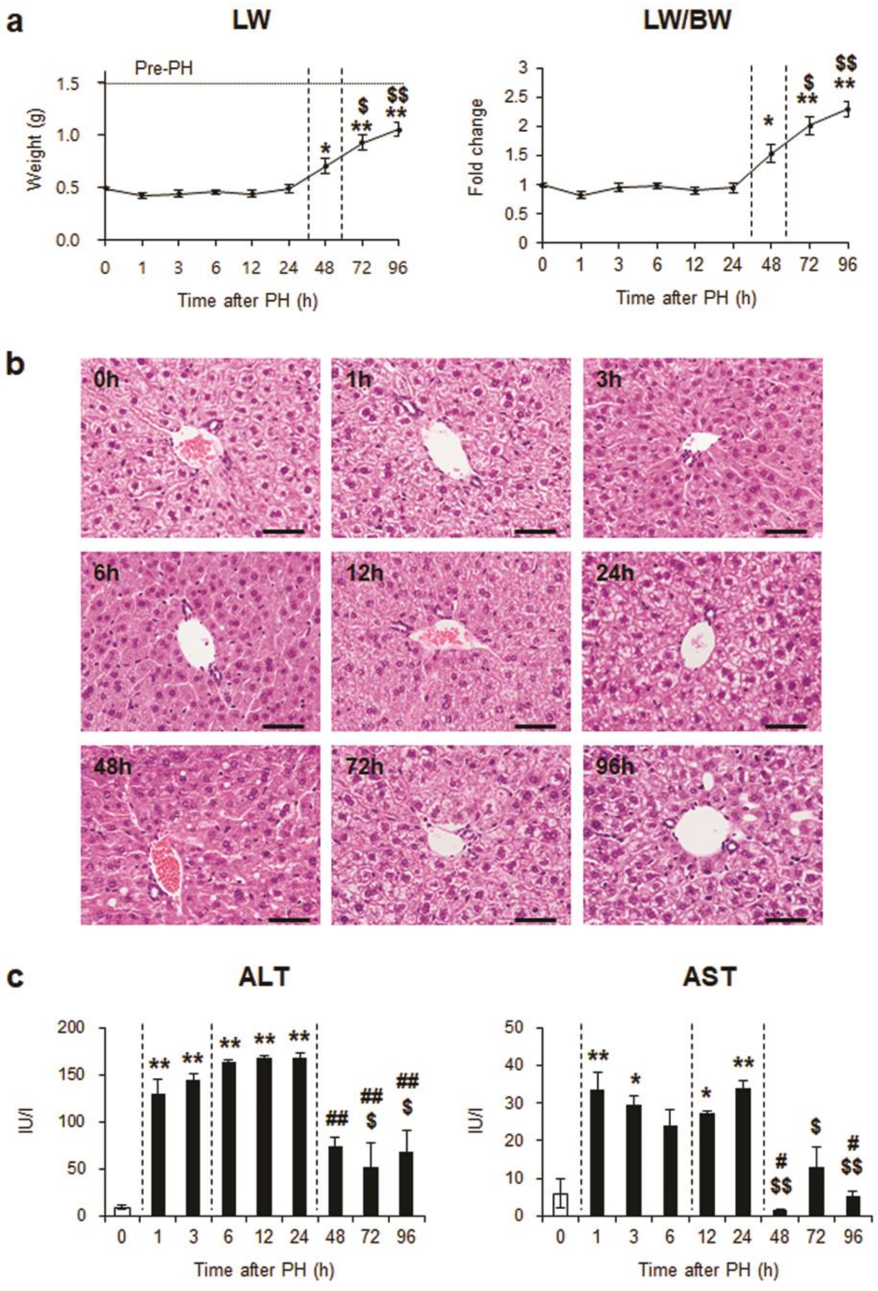

Supplementary Figure S2

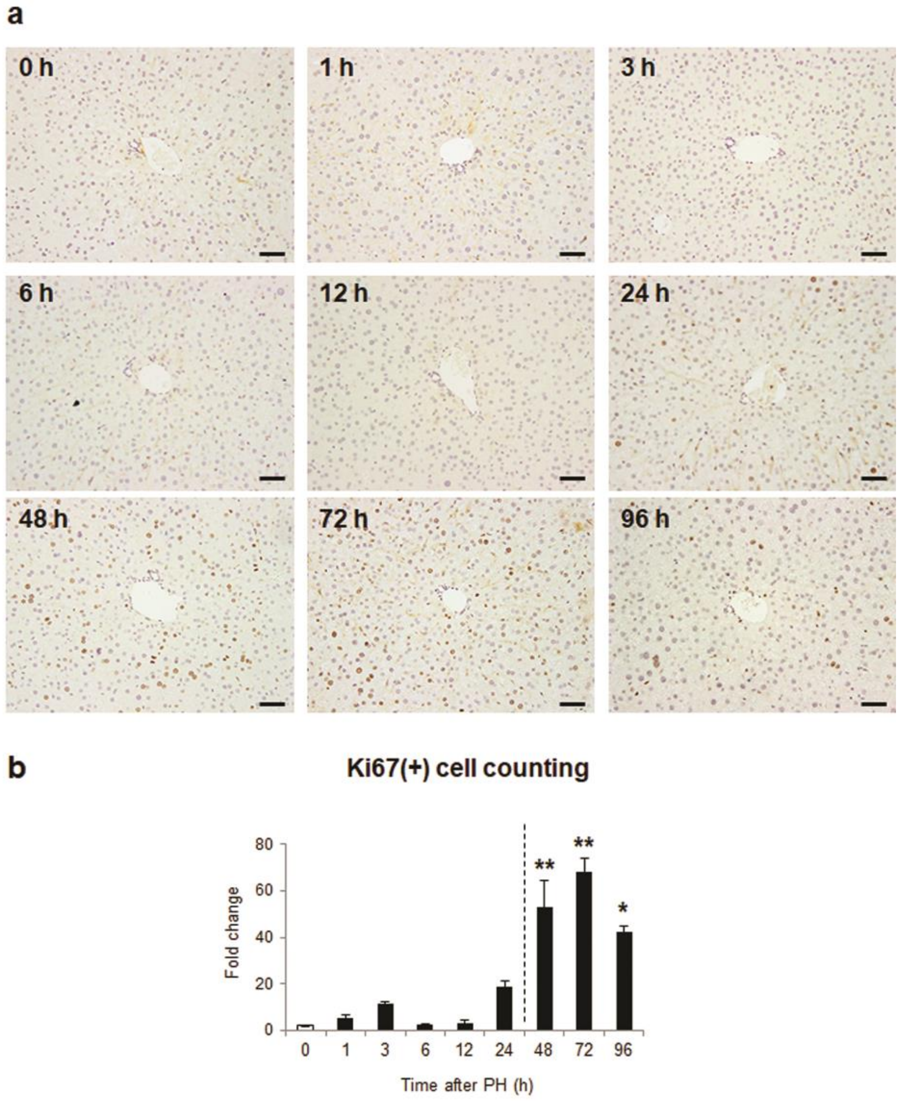

Supplementary Figure S3

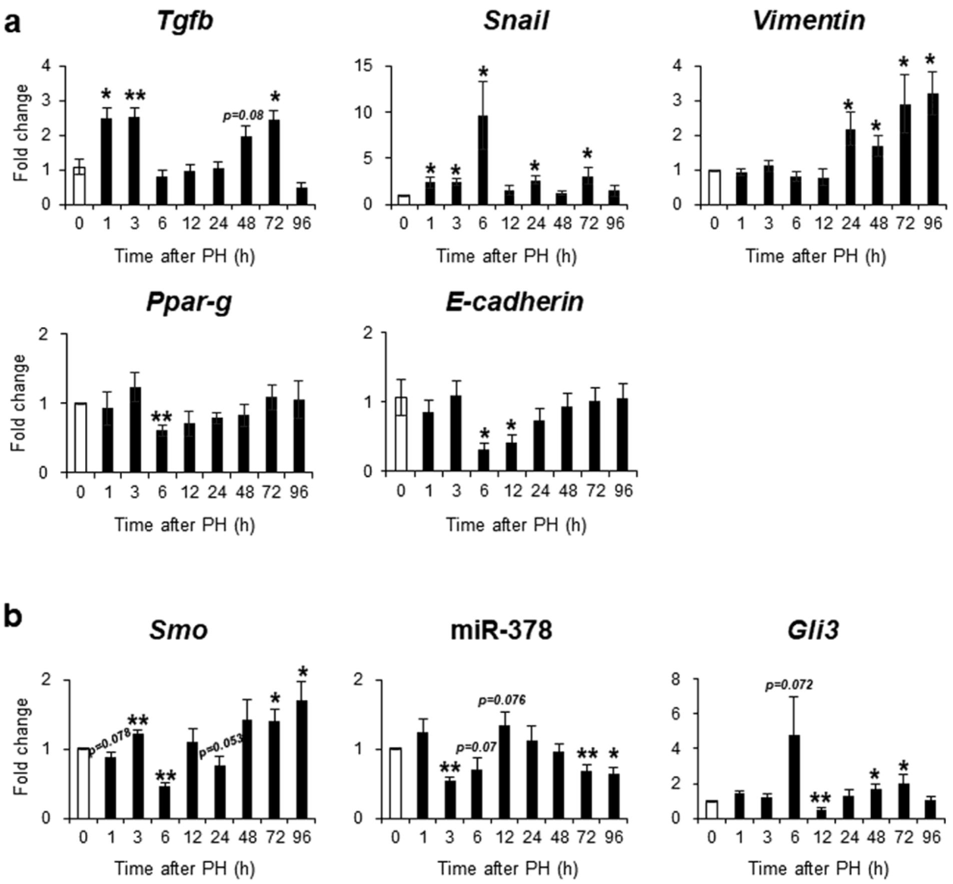

Supplementary Figure S4

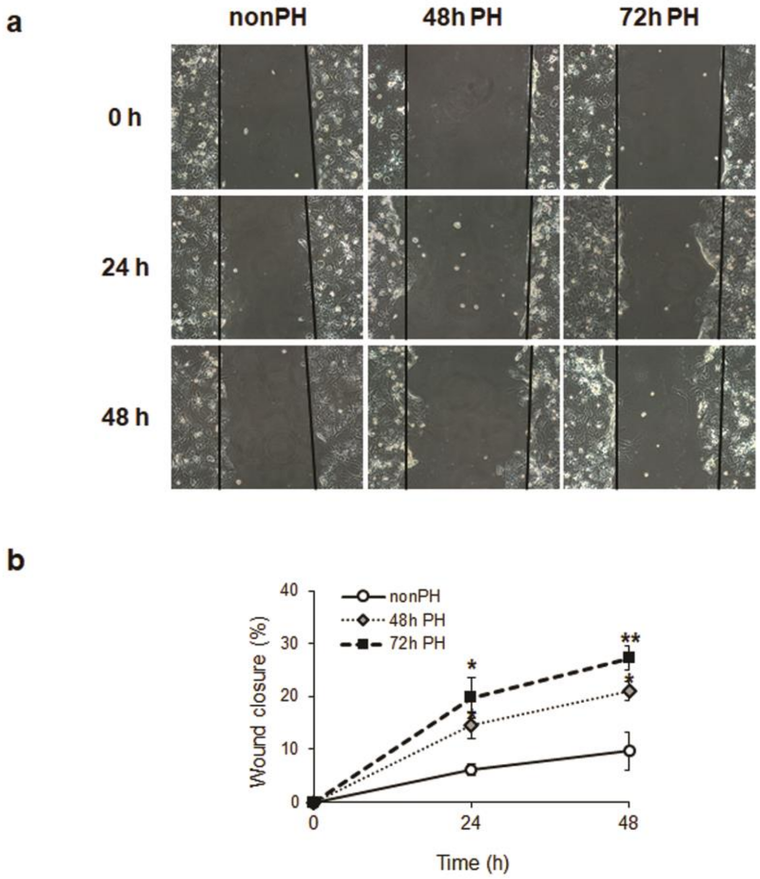

97 **Supplementary Figure S5**

98

99

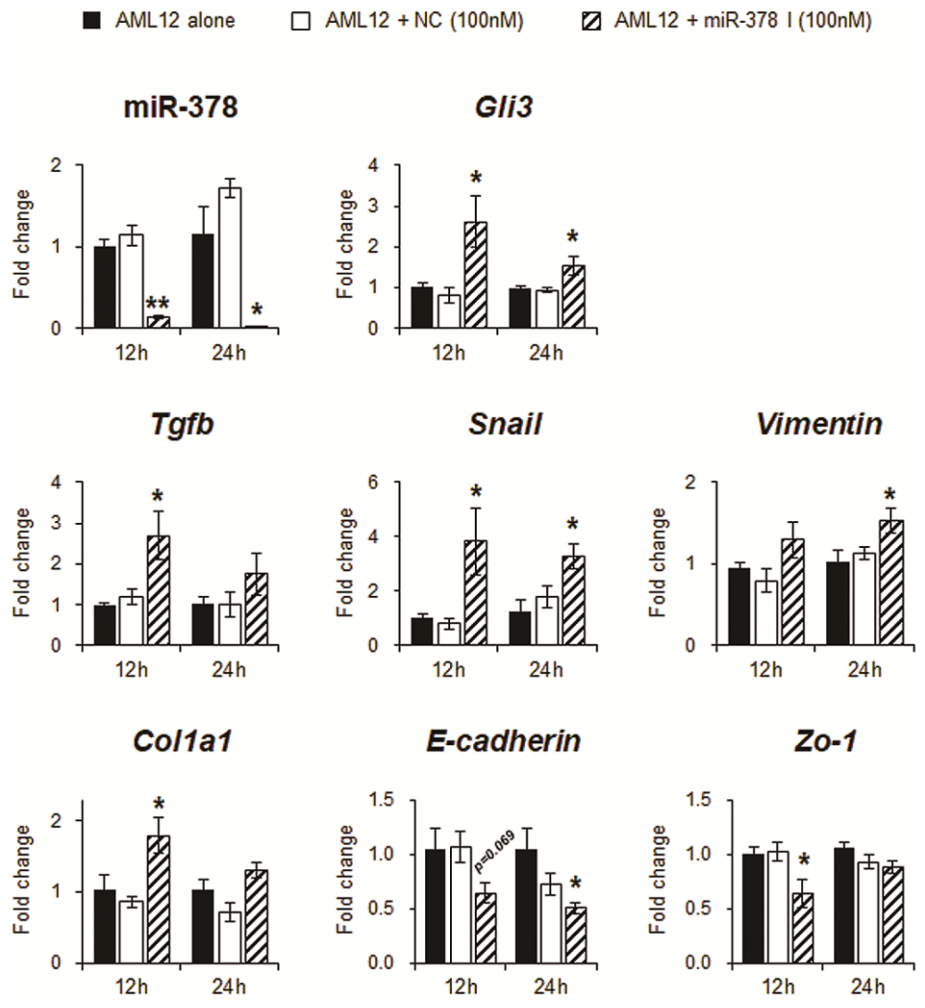

Supplementary Figure S6

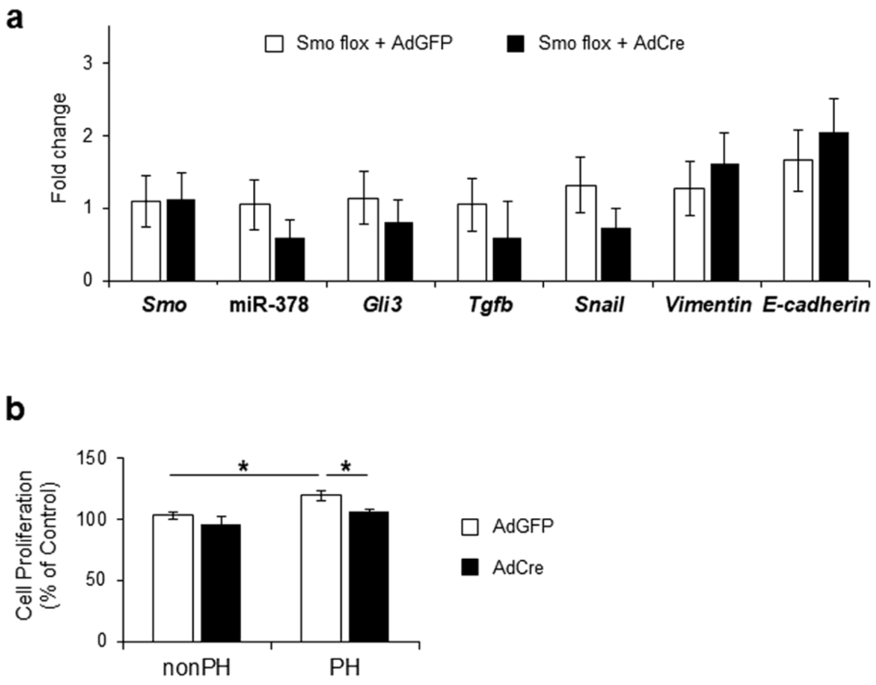

Supplementary Figure S7

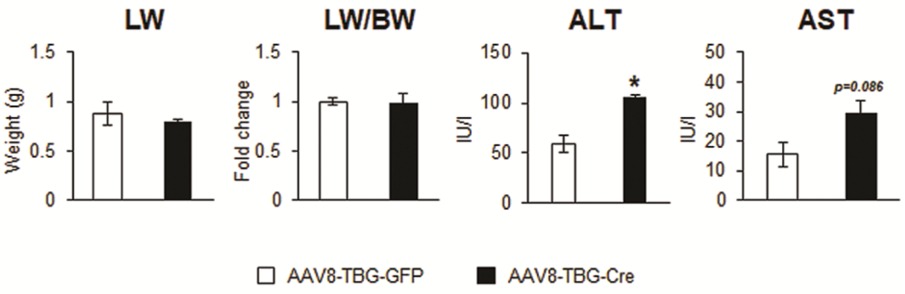

Supplement: Supplementary file 1 — Supplementary Information (marked-up ver) [file 41419_2018_762_MOESM1_ESM.pdf]
